# Development of Luciferase Immunoprecipitation Systems (LIPS) Assay to Detect IgG Antibodies against Human Respiratory Syncytial Virus G-Glycoprotein

**DOI:** 10.3390/vaccines7010016

**Published:** 2019-02-01

**Authors:** Roberta Lynne Crim, Sangeeta Kumari, Priyanka Jayanti, Susette Audet, Ashwin Kulkarni, Judy Beeler

**Affiliations:** Office of Vaccines Research and Review, CBER, FDA, Silver Spring, MD 20993, USA; roberta.crim@fda.hhs.gov (R.L.C.); sk4px@virginia.edu (S.K.); pjayanti94@gmail.com (P.J.); susette.audet@fda.hhs.gov (S.A.); schwinn6@gmail.com (A.K.)

**Keywords:** respiratory syncytial virus, immune responses, G protein

## Abstract

Respiratory syncytial virus (RSV) causes severe lower respiratory tract disease in infants and the elderly. Although there is no licensed vaccine, RSV-F and -G glycoproteins are targets for vaccine development and therapeutics. We developed an assay that can detect anti-RSV-G IgG antibodies, either as a biomarker of natural exposure or immunization. RSV genes encoding native and mutated G (mG) proteins from subgroups A and B strains were cloned, expressed as luciferase-tagged proteins, and tested individually to detect anti-RSV-G specific IgG antibodies using a high-throughput luciferase immunoprecipitation system (LIPS-G). RSV monoclonal antibodies and polyclonal antisera specifically bound in the LIPS-G_A_ and/or -G_B_ assays; whereas anti-RSV-F and -N, and antisera against measles virus or human metapneumovirus did not bind. Anti-RSV-G_A_ and -G_B_ IgG responses detected in mice infected intranasally with RSV-A or -B strains were subtype specific. Subtype specific anti-RSV-G_A_ or -G_B_ IgG responses were also detected using paired serum samples from infants while human adolescent serum samples reacted in both LIPS-G_A_ and -G_B_ assays, reflecting a broader experience.

## 1. Introduction

Respiratory syncytial virus (RSV) is the most common cause of serious lower respiratory tract infection in young children worldwide and a frequent cause for hospitalization in children less than 2 years old in the USA. Severe infections early in life may predispose to asthma later. RSV also impacts the elderly and can be especially debilitating in those with underlying heart or lung disease and accounts for nearly as many hospitalizations as influenza [1,2]. Currently, there are no vaccines licensed to prevent RSV infection, despite more than 50 years of effort. An FDA approved humanized anti-RSV-F monoclonal antibody prevents severe RSV disease but is only approved for use in pre-term and high-risk infants [3]. The development of an effective RSV vaccine has proven elusive, in part due to the immature immune system of infants and immune senescence of aged adults. Maternal antibodies may further complicate the immune response to RSV infection in young infants by suppressing the initial response to viral infection allowing reinfection to occur [4]. While it is now commonly accepted that most neutralizing antibodies target pre-F protein, there is also evidence demonstrating that both RSV-pre-F and -G antibodies can modulate RSV disease severity in young infants [5,6]. Additionally, RSV-G antibodies are under evaluation for their role in complement-mediated antiviral activity, blockade of airway epithelial cell infection, and anti-inflammatory activity [7]. Accordingly, assays that detect anti-RSV-G antibodies can be used to evaluate antibody responses elicited either by immunization with G-containing vaccines or by natural infection to assess incidence rates during the RSV season.

RSV is categorized into two distinct antigenic subgroups, RSV-A and RSV-B, which may co-circulate or independently dominate during yearly epidemics. The RSV-F protein is most conserved (>90%) between RSV-A and -B strains, thus making this protein an ideal target for vaccine design and therapeutics. In contrast, RSV-G protein is more variable across subgroups (53% conservation at the amino acid level), although there is a central conserved domain (CCD) region that is 100% conserved amongst all circulating strains. This central conserved region is essential for infectivity in vivo, since it mediates attachment to airway epithelial cells, and plays a role in the host immune response due to the resemblance to the CX3C chemokine motif [8,9]. The CCD region of the RSV-G protein has been targeted for monoclonal antibody development and RSV-G based vaccines; however, areas outside the CCD region may also play a part in protection from infection [7,10,11,12,13,14,15,16].

During clinical trials of candidate vaccines, RT-PCR assays are often used for case confirmation and to identify RSV-infected subjects. While these assays can be very sensitive, the RSV-infected cohort may sometimes be underestimated due to logistics involved in sample collection. Serological assays have been used to complement and support RT-PCR testing to identify the RSV-exposed population. Serological assays enhanced detection of RSV cases by almost 12% over RT-PCR methods alone [17]. Unfortunately, many of the currently available serological tests that detect anti-RSV IgG responses use lysates of RSV-infected cells, purified virus, or vaccine antigens to bind antibodies. These tests do not provide information about the subtype of the infecting strain and may not discriminate between responses elicited by vaccine from those seen following natural infection. In contrast, serological assays using non-vaccine, off-target antigens have successfully identified individuals undergoing RSV infection during the post-vaccination period [18,19]. The immunoprecipitation assay described in this report uses two luciferase-tagged RSV antigens (RSV-G_A_ and RSV-G_B_) to help fill the gap in serology testing. These assays can be used to detect anti-RSV- G IgG specific responses as a tool for RSV surveillance to detect virus exposures among subjects given vaccines that contain or express F-only antigen. 

## 2. Materials and Methods

### 2.1. Cells and Viruses

HEp2, Vero, and COS-1 cells were grown as previously described [20,21,22]. RSV-A2 and -18537 were obtained from Drs. Murphy and Chanock and prepared in HEp2 cells as previously described [23]. RSV-B1, obtained from Dr. Fran Rubin (NIAID, NIH, Bethesda, MD, USA, was amplified in Vero cells after receipt to generate a working pool. Measles virus (MV, from Dr. Paul Albrecht, FDA, Silver Spring, MD, USA and human metapneumovirus (hMPV, from Dr. Ursula Buchholz, NIH) were grown in Vero cell in a similar manner. Viruses were sucrose gradient purified and UV-inactivated prior to animal immunizations as previously described [22,24]. Virus inactivation was confirmed by culture on HEp2 cell monolayers for six days with daily observation for cytopathic effect.

### 2.2. Antibodies and Sera 

Murine IgG monoclonal antibodies (MAbs) against RSV-G (1227, 1197, 104-5, 109), RSV-F (1243), PIV3-F (b108), or PIV3-HN (170) were obtained from a WHO Reagent Bank. Murine MAb’s 131-2G (anti-RSV-G), 130-12H (anti-RSV-N), and anti-FLAG were purchased from Millipore-Sigma (St. Louis, MO, USA). 

Four-to six-week-old female BALB/c mice were infected intranasally with 0.1 mL inoculum containing approximately 10^6^ TCID_50_ of RSV-A2 or RSV-B1 on days 0 and 42. Sera were collected before infection and on days 35 and 77. Pre-immune mouse sera served as the negative control. Animal experiments were approved by the U.S.A. FDA Institutional Animal Care and Use Committee (IACUC) (protocol #2010-24).

Rabbits were immunized with UV-inactivated, sucrose-gradient purified viruses (RSV-A2, RSV-18537) or proteins (RSV-F_A_, RSV-G_A_) as previously described [25]. Another group of rabbits were immunized with UV-inactivated viruses including MV and hMPV, in a similar manner. Rabbits immunized with purified UV-RSV-18537 were also boosted once with 0.12 mg of purified RSV-G_B_ protein. Rabbits were also immunized intramuscularly with 400 µg Keyhole limpet hemocyanin (KLH)-conjugated synthetic linear peptides: G_A231-250_ (RSV-A amino acids T_231_-S_250_), G_A262-291_ (RSV-A amino acids M_262_-S_291_), LHWT-G_A_ or LHWT-G_B_ (CX3CR1 binding domain consisting of V_171_-P_202_ for both RSV-A and -B strains) [26]. Immunoprecipitation assays evaluating reactivity of rabbit antisera with luciferase-tagged RSV-G proteins used rabbit anti-FLAG IgG (Sigma-Aldrich, St. Louis, MO, USA) and pre-immune rabbit sera as positive and negative controls, respectively. Animal experiments were approved by the U.S.A. FDA IACUC (protocol #2001-23).

Anonymous human serum samples (6 mo to 14 yrs) were obtained with informed consent from Institutional Review Board (IRB)-approved studies and tested with approval by the FDA Research involving Human Subjects Committee (FDA RIHSC #11-030B). Thirty-six (of 100) samples obtained at 9 months were previously identified as sero-negative for both anti-RSV neutralizing and anti-RSV-N IgG antibodies [25]. Of these 36, 12 had paired samples obtained at 15–18 months of age; these 12 paired serum samples were further evaluated herein. Human reference immune globulin to RSV (BEI NR-21973) was diluted to 1% IgG concentration in DPBS, unless otherwise stated. Human sera depleted of IgG, IgM and IgA (IgΔ) was obtained from SunnyLab (Sittingbourne, UK). Aliquots of all reference sera were stored at −20 °C. 

### 2.3. Generation of Renilla Luciferase-Tagged RSV-A2 and RSV-B1 G/mG Protein Constructs

A mammalian expression vector, pREN2, was previously described and was used to generate *Renilla* luciferase (Ruc)-tagged RSV-G_A_, RSV-mG_A_, RSV-G_B_, or -RSV-mG_B_ proteins (Ruc-G_A_, Ruc-mG_A_, Ruc-G_B_, or Ruc-mG_B_) [21]. Each construct contained both a FLAG epitope and Ruc-tag at the amino terminus of the G protein. The RSV-G_A_ and -G_B_ genes were amplified from HEp2 infected RSV-A2 (GeneBank accession no. M11486.1) or RSV-B1 (GeneBank accession no. AF013254.1), respectively, using the primers provided in Table 1. The RSV-A2 and -B1 DNA were cloned in TOPO plasmids and then sub-cloned into pREN2 downstream of *Renilla* luciferase sequence between *EcoRI* and *Xho1* restriction sites as previously described [25].

Synthetic DNA constructs representing a mutated form of G protein (mG) were purchased (Genscript, Piscataway, NJ, USA). Genbank sequences M11486.1 and AF013254.1 were used to design RSV-A2 and -B1 mG constructs, respectively, and included amino acid substitutions in both constructs at positions 48 (M48I) and 49 (I49V) to abolish the secreted form of G [27]. Synthetic constructs were amplified, restriction enzyme digested, gel purified, and then cloned into pREN2 as described above. The sequence and integrity of all DNA constructs were confirmed using automated DNA sequencing. 

### 2.4. Renilla Luciferase (Ruc) RSV-G_A_ and RSV-G_B_ Expression 

COS-1 cell monolayers were transfected with pREN2 (without insert), pREN2+RSV-G_A_, or pREN2+RSV-G_B_ as previously described [28]. Expression of luciferase-tagged native Ruc-G proteins were confirmed by Western Blot following immunoprecipitation with murine anti-FLAG-coated Sepharose beads (Clontech, Mountain View, CA, USA) per the manufacturer’s protocol. Precipitated proteins were separated by SDS-PAGE prior to detection in Western blot. Blots were probed with the following rabbit anti-sera: anti-FLAG, anti-G_A_ protein or anti-LHWT-G_B171-202_ peptide followed by detection with goat anti-rabbit horseradish-peroxidase and LumiGlo chemiluminescent substrate. Expression of Ruc-mG proteins was similarly confirmed.

### 2.5. LIPS-G Assay to Detect RSV-G Specific IgG Antibodies

COS-1 cell lysates containing Ruc only (without insert), Ruc-G_A_, Ruc-mG_A_, Ruc-G_B_, or Ruc-mG_B_ were obtained and used in the luciferase immunoprecipitation system (LIPS) assay as previously described except samples bound to Sepharose beads were washed four times with PBS [25]. Briefly, Ruc-tagged antigen was diluted to 10^7^ *Renilla* light units (RLU) in 50 µL per well and mixed with 50 µL of diluted antibody or serum for 1h at room temperature on a rotary shaker. Antigen-antibody mixtures were then transferred to 96-well high-throughput sequencing (HTS) filter plates containing 5 µL of a 30% suspension of Ultralink protein A/G beads and further incubated for 1h at room temperature with shaking. Filter plates were washed and RLU content determined following the addition of coelenterazine (Promega, Madison, WI, USA) in a SpectraMax L luminometer. *Renilla* light units obtained from triplicate wells were adjusted by subtracting the mean of the blank wells and then averaged. A positive value was ≥5 standard deviations above the mean value of the negative control, as recommended [28]. To allow for comparison of reactivity between antigens, data were normalized based on reactivity seen with anti-FLAG antibody against each antigen.

### 2.6. Production and Purification of Recombinant RSV Proteins in E. Coli for Competition Assays and Immunizations 

Purified recombinant RSV-N, RSV-G_A_, and RSV-G_B_ proteins used for competition assays were produced using nickel chromatography as previously described [25]. Generation of RSV-G_B_ protein used for rabbit immunization was performed as previously described with slight modification [29]. Imidazole and other small molecules were removed from the concentrated purified pET-RSV-G_B_ protein with a Zeba desalting column (Thermo Fisher, Waltham, MA, USA) followed by 0.22 µm sterile filtration. The sterile purified pET-RSV-G_B_ protein was endotoxin free. 

### 2.7. Competition Assay Using Recombinant RSV-G_A_ or RSV-G_B_ Protein

Human immune globulin (huIgG, BEI NR-21973) was diluted to 0.1% using sterile water. For the competition, 40 µL of recombinant RSV-G_A_, RSV-G_B_, or RSV-N protein (0.2 mg/mL) were mixed with 10 µL of 0.1% huIgG (BEI NR-21973). Mixtures were incubated for 1hr and then 50 µL of *Renilla* tagged antigen (1 × 10^7^ RLU) added prior to testing in the LIPS assay as described above in Section 2.5. Control wells contained 0.1% huIgG (BEI NR-21973) in the absence of purified RSV protein. 

### 2.8. Determination of Limit of Detection

Human immune globlulin (huIgG, BEI NR-21973) was tested in triplicate at concentrations ranging from 10% to 0.000001% IgG (100, 10, 1, 0.1, 0.01, 0.001, 0.0001 mg/mL total IgG) in parallel with IgΔ. Serum and immune globulin samples were also tested using a plaque reduction neutralization (PRN) assay versus RSV-A2 or RSV-B1, as previously described [22]. Fifty percent endpoint titers (Neutralizing Dose, ND_50_) were calculated using the Spearman-Kärber method [30].

### 2.9. Statistical Analysis

A two-tailed *t* test was used to compare mean log_10_ RLU values obtained in each assay using human adolescent serum samples.

## 3. Results

### 3.1. Expression of Ruc-G_A_ and Ruc-G_B_ Protein in COS-1 Lysates

**Figure 1:**
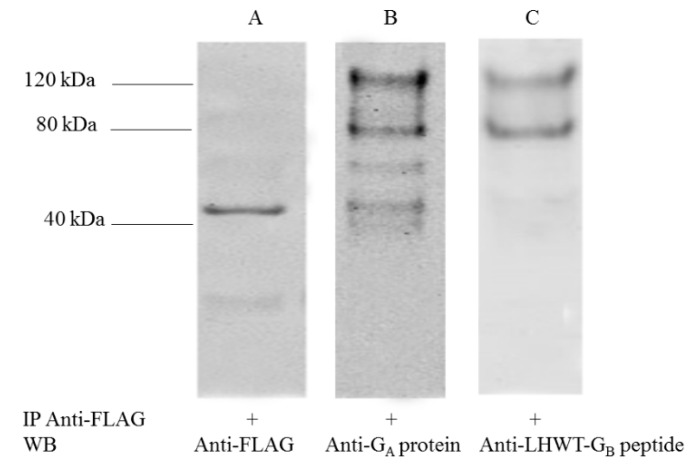
Expression of Ruc-G antigens. Ruc-tagged antigens were immunoprecipitated using beads coated with anti-FLAG IgG and evaluated using Western blot assay as described in materials and methods to confirm expression of Ruc (no insert) (lane **A**), Ruc-G_A_ (lane **B**), and Ruc-G_B_ (lane **C**) antigens in COS-1 lysates using rabbit antisera for detection. Molecular weight markers are noted.

Expression of Ruc (no insert), Ruc-G_A_, and -G_B_ proteins was confirmed in Western blot following immunoprecipitation of proteins obtained from COS-1 lysates transfected with pREN2, pREN2+RSV-G_A_, or pREN2+RSV-G_B_, respectively. Protein bands at 120kDa and ~80kDa were observed when blots were probed with rabbit anti-G_A_ protein (Figure 1B) or rabbit anti-LHWT-G_B171-202_ (Figure 1C), consistent with full length, fully-processed Ruc-tagged RSV-G (120kDa) and unglycosylated or N-glycosylated RSV-G tagged with Ruc antigen (80 kDa). As expected, Ruc antigen without insert was detected by rabbit anti-FLAG at ~40 kDa in the COS-1 lysate transfected with pREN2 only (Figure 1, lane A). Rabbit pre-immune serum was negative against all antigens. Results were confirmed by probing the same antigens with rabbit anti-*Renilla* luciferase antibody. Likewise, RSV-G mutated proteins (i.e., Ruc-mG_A_ and Ruc-mG_B_) gave reactivity patterns similar to those seen using Ruc-G_A_ and Ruc-G_B_ antigens. 

### 3.2. Assay Specificity 

**Figure 2:**
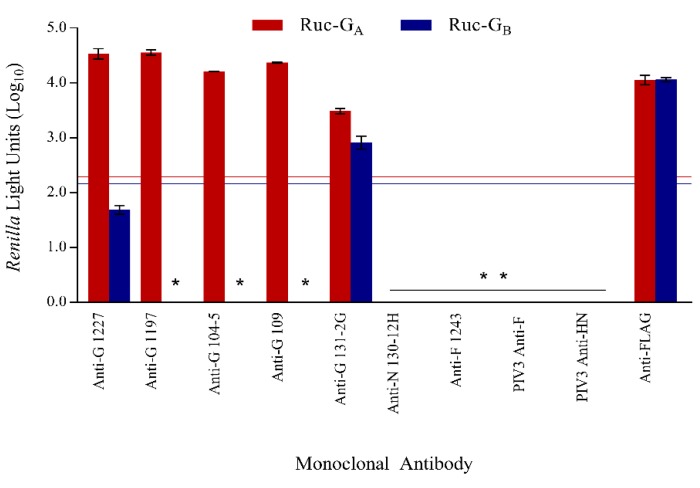
Specificity of the LIPS-G assays. Specificity of respiratory syncytial virus (RSV) LIPS-G assays in detecting anti-RSV-G IgG antibodies was shown using murine monoclonal antibodies. The mean reactivities against Ruc-G_A_ and Ruc-G_B_ antigens are shown in red and blue bars, respectively, and error bars represent standard deviations. Data were normalized against murine anti-FLAG antibody. Cutoff values for a positive signal were five standard deviations above the mean value of all four negative controls and are shown as horizontal red (Ruc-G_A_) and blue lines (Ruc-G_B_). * Represents a negative result.

The ability of the LIPS-G_A_ and LIPS-G_B_ assays to detect anti-RSV-G IgG specific binding was evaluated using murine monoclonal antibodies (Figure 2). RSV anti-G MAbs 1227, 1197, 104-5, and 109 bound strongly in the LIPS-G_A_ assay only and did not bind to Ruc-G_B_ antigen. RSV anti-G MAb 131-2G, known to be reactive with both RSV-G_A_ and -G_B_ proteins, bound in both assays. MAbs directed against other viral antigens, including RSV-N (130-12H), RSV-F (1243), PIV3-F (b108), and PIV-HN (170), were negative in both the LIPS-G_A_ and LIPS-G_B_ assays, while the anti-FLAG antibody bound both antigens. Cutoff values were determined using the mean value obtained for all four negative antibodies (130-12H, 1243, b108, and 170) and were set at 2.29log_10_ RLU (red line) and 2.16log_10_ RLU (blue line) for Ruc-G_A_ and Ruc-G_B_, respectively. All MAbs were negative when tested against Ruc only antigen without insert.

**Figure 3:**
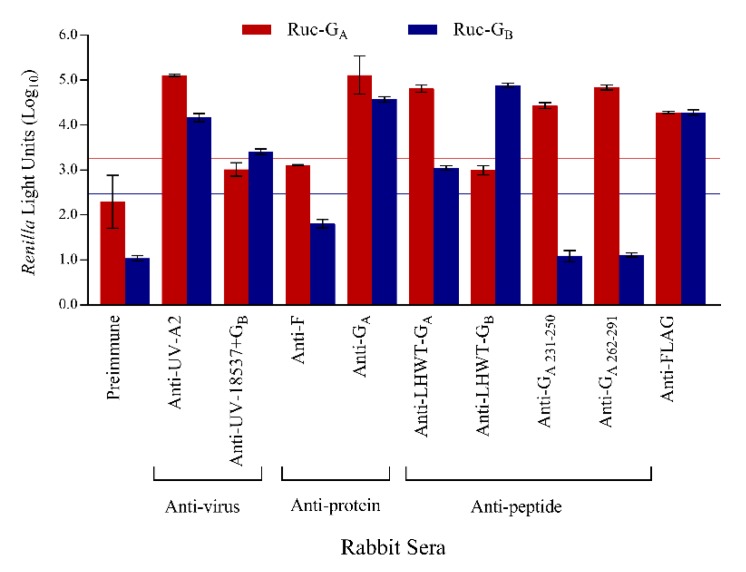
Specificity of the LIPS-G assays. Specificity of RSV LIPS-G assays was also shown using rabbit antisera. Mean reactivities against Ruc-G_A_ and Ruc-G_B_ antigens are shown in red and blue bars, respectively, and error bars represent standard deviations. Data were normalized against rabbit anti-FLAG antibody. Cutoff values for a positive signal were five standard deviations above the mean value of the negative control and are shown as horizontal red (Ruc-G_A_) and blue lines (Ruc-G_B_).

Antisera from rabbits immunized parenterally with RSV-A or -B viruses or antigens were tested in the LIPS-G_A_ and -G_B_ assays to determine if binding was subtype specific or cross-reactive with the heterologous protein (Figure 3). Rabbit anti-RSV-A2 and anti-RSV-G_A_ glycoprotein were strongly positive when tested against Ruc-G_A_ antigen with a lower but positive signal when tested against Ruc-G_B_ antigen. In contrast, antisera from rabbits immunized with RSV-18537 and boosted with purified G_B_ protein were only positive in LIPS-G_B_ assay (cutoff = 2.47log_10_ RLU). Interestingly, subtype specific binding was noted using antisera from rabbits immunized with synthetic RSV-G_A_ peptides representing amino acids 231-250 or 262-291 since these antisera were positive against Ruc-G_A_ antigen but negative when tested using the heterologous Ruc-G_B_ antigen. The amino acids within the central region of the RSV-G primary sequence are highly-conserved and are thought to mediate an important first step in RSV binding to ciliated lung epithelial cells. Accordingly, we expected to see cross-reactivity in antibody responses raised against peptides that represent the RSV-G_A_ versus RSV-G_B_ central conserved domains. Anti-LHWT-G_A171-202_ was clearly positive against Ruc-G_A_ antigen, and also bound to Ruc-G_B_ antigen, albeit less well. Surprisingly, reciprocal cross-reactivity was not observed with anti-LHWT-G_B171-202_ since it was reactive only against Ruc-G_B_ antigen and did not bind Ruc-G_A_ antigen. Pre-immune serum did not react with either Ruc-G_A_ or Ruc-G_B_ antigens while rabbit anti-FLAG reacted positively in both assays. Rabbit antisera to MV or hMPV were negative against all antigens tested. All rabbit anti-sera were negative when tested against the Ruc only antigen while the anti-FLAG antibody gave positive results as expected.

### 3.3. Sensitivity of LIPS-G Assays in Detecting Anti-RSV-G IgG Antibodies Compared to PRN

**Figure 4:**
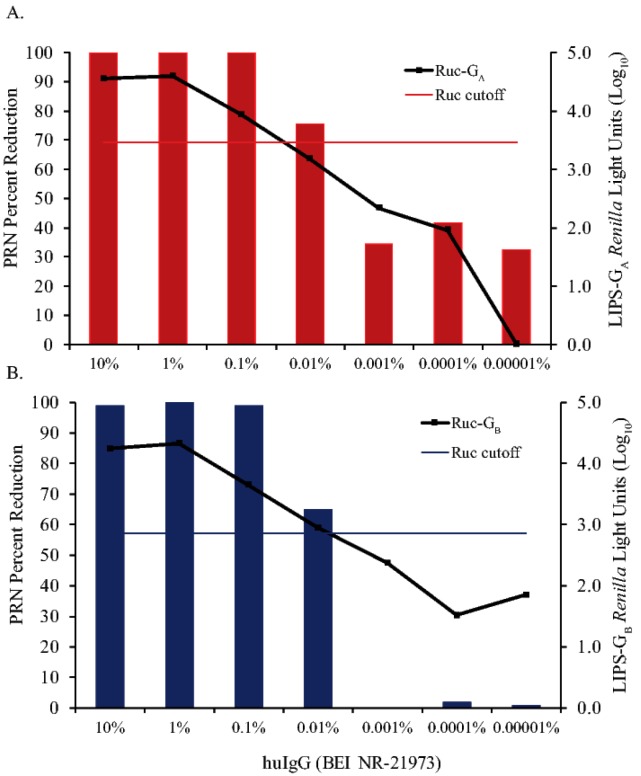
Detection of anti-RSV antibodies using LIPS-G_A_ and -G_B_ assays versus plaque reduction neutralization (PRN) testing. Human immune globulin (huIgG, BEI NR-21973) was used neat or serially diluted 10-fold into IgΔ from 10% to 0.00001% IgG. Samples were tested in triplicate in immunoprecipitation assays and in duplicate in plaque reduction neutralization assays against Ruc-G_A_ antigen and RSV-A2, respectively (panel **A**) or against Ruc-G_B_ antigen and RSV-B1, respectively (panel **B**). The results of testing in the immunoprecipitation assay are shown by the black lines in each figure. Cutoff values for a positive result in the immunoprecipitation assays were five standard deviations above the mean value of the negative control (IgΔ) and are shown as horizontal red (Ruc-G_A_) and blue lines (Ruc-G_B_). In the PRN test, a reduction in the mean plaque count of ≥50% (in wells containing huIgG) relative to the mean plaque count seen in virus control wells (in the absence of huIgG) was considered significant.

Human immune globulin (huIgG, BEI NR-21973) was tested over a wide range of IgG concentrations in the LIPS-G_A_, LIPS-G_B_, and PRN assays to compare sensitivities in detecting anti-RSV antibodies. Anti-RSV-G specific IgG binding was detected at concentrations as low as 0.1% (1.0 mg/mL total IgG) in LIPS-G_A_ (Figure 4A) and 0.01% (0.1 mg/mL total IgG) in LIPS-G_B_ (Figure 4B) assays. PRN antibodies capable of inhibiting RSV infectivity by 50% or more were detected at concentrations as low as 0.01% (0.1 mg/mL total IgG) for both RSV-A2 and RSV-B1 viruses. No binding was seen at any dilution when huIgG was tested against Ruc antigen alone and Ig∆ did not inhibit RSV in the PRN assay. Cutoffs for a positive response in the LIPS-G_A_ and -G_B_ assays were determined using the mean values obtained for the tests on IgΔ in each assay (3.46log_10_ RLU and 2.86log_10_ RLU for Ruc-G_A_ and Ruc-G_B_, respectively). 

### 3.4. Confirmation of Specificity

**Figure 5:**
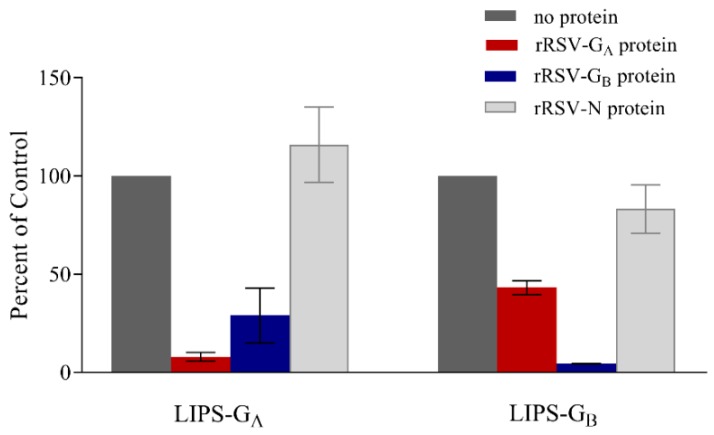
Purified RSV-G proteins inhibit binding of human immune globulin (huIgG) in LIPS-G assays. Soluble purified recombinant rRSV-G_A_ (red), rRSV-G_B_ (blue), or rRSV-N (light gray) proteins were incubated with an equal volume of huIgG diluted to 0.1%. Binding of huIgG in the absence of competitor protein (dark gray) was set at 100% in each assay.

After confirmation of huIgG binding, specificity was further demonstrated by evaluating the ability of recombinant purified proteins to block binding of huIgG in each LIPS-G assay. When huIgG was incubated with recombinant rRSV-G_A_ protein prior to testing in the LIPS-G_A_ and -G_B_ assays, binding was decreased 92% (i.e., 8% of control) and 57% (i.e., 43% of control) using Ruc-G_A_ and Ruc-G_B_ antigens, respectively. Likewise, following incubation with recombinant rRSV-G_B_ protein, binding of huIgG was decreased 71% (i.e., 29% of control) and 96% (i.e., 4% of control) in LIPS-G_A_ and LIPS-G_B_ assays, respectively (Figure 5). Pre-incubation with recombinant RSV-N protein did not block binding of huIgG in either assay. 

### 3.5. LIPS-G Assays Detect Antibodies Elicited by Natural Infection

**Figure 6:**
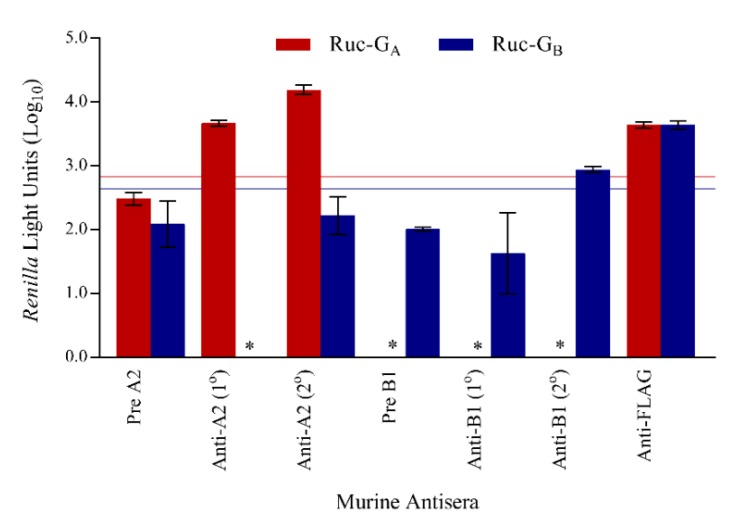
Detection of subtype-specific, anti-RSV-G IgG antibody responses. Murine immune responses were evaluated following primary and secondary intranasal infection with RSV-A2 or -B1. The mean reactivities against Ruc-G_A_ and Ruc-G_B_ antigens are shown in red and blue bars, respectively, and error bars represent standard deviations. Data were normalized against murine anti-FLAG antibody. Cutoff values for a positive signal were five standard deviations above the mean value of the negative control and are shown as horizontal red (Ruc-G_A_) and blue lines (Ruc-G_B_). * Represents a negative result.

Murine immune responses following sequential infections with either RSV-A2 or RSV-B1 viruses were evaluated using LIPS-G_A_ and -G_B_ assays (Figure 6). RSV-A2 infected mice undergoing primary infection developed a subtype-specific anti-G_A_ IgG immune response that was boosted following a second infection. In contrast, mice infected once with RSV-B1 failed to develop an anti-G IgG antibody response detected using either LIPS-G_A_ or LIPS-G_B_ assays and only responded weakly with a subtype-specific IgG response following the second exposure to RSV-B virus. Sera from mice obtained prior to RSV infection were negative in all assays and values obtained from these tests were used to set the cutoffs for a positive result in each test (2.83log_10_ RLU and 2.64log_10_ RLU for LIPS-G_A_ and LIPS-G_B_, respectively). Murine anti-FLAG antiserum gave a positive result as expected. Murine antisera did not bind to the Ruc-only antigen.

**Figure 7:**
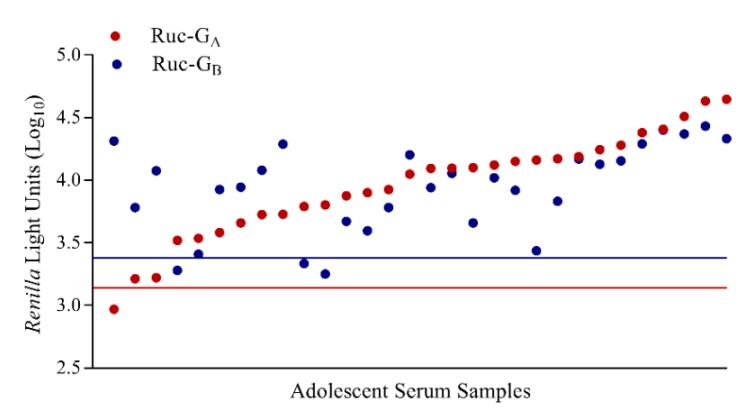
Detection of anti-RSV-G IgG antibody responses in human adolescent serum samples in LIPS-G_A_ and LIPS-G_B_ assays. Samples from 30 adolescent children, 10–14 years of age, were tested and data were normalized against reactivity of rabbit anti-FLAG antibody in each assay. Immunoglobulin depleted serum (IgΔ) was used as the negative control and cutoff values are shown as horizontal red (Ruc-G_A_) and blue lines (Ruc-G_B_). Results are ranked from low to high based on testing against Ruc-G_A_ (red circle) and paired with the corresponding result for each subject against Ruc-G_B_ (blue circle) antigen.

Similarly, human serum samples from 30 adolescent children, 10–14 years of age, were tested in LIPS-G_A_ and -G_B_ assays (Figure 7). Serum samples were also tested in PRN assays and all samples had detectable neutralizing antibodies against both RSV-A2 (ND_50_ range 1:35 to 1:10,000) and RSV-B1 (ND_50_ range 1:31 to 1:1795). Human IgΔ was the negative control and used to set the cutoff for a positive result (cutoffs were 3.14log_10_ RLU for Ruc-G_A_ and 3.38log_10_ RLU for Ruc-G_B_). Overall, 29 and 27 samples (out of 30) had detectable IgG antibodies when tested against Ruc-G_A_ and Ruc-G_B_ antigens, respectively. No correlation was seen when we compared the results of the LIPS testing with PRN assay results by RSV subtype. 

### 3.6. LIPS-G Assays Detect Anti-RSV-G IgG Specific Responses in Paired Sera from Young Children

**Figure 8:**
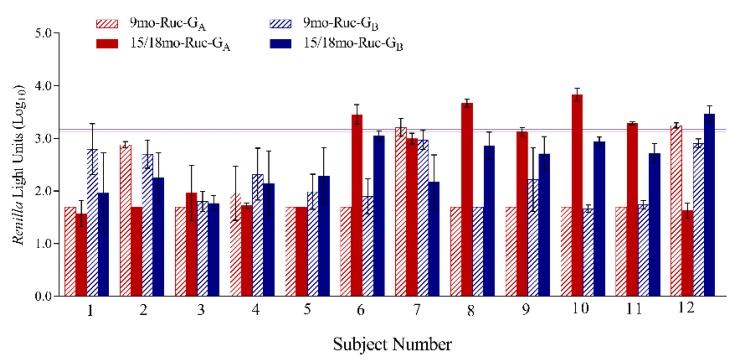
Twelve paired serum samples obtained at 9 and 15/18 months of age were tested against Ruc-G_A_ and Ruc-G_B_ antigens. The mean reactivities for individual samples tested against Ruc-G_A_ and Ruc-G_B_ antigens are shown in red and blue bars, respectively, and error bars represent standard deviations. Cutoff values for a positive signal were five standard deviations above the mean value of the negative control and are shown as horizontal red (Ruc-G_A_) and blue lines (Ruc-G_B_).

A subset of 12 paired sera, collected at 9 months and then again at 15–18 months, was tested in both the LIPS-G_A_ and LIPS-G_B_ assays (Figure 8). This subset was previously identified as negative for RSV antibodies at 9 months of age, but had detectable RSV neutralizing and anti-RSV-N IgG antibodies at 15–18 months of age, suggesting an RSV exposure had occurred in the interval between sampling. At 9 months, 10 of 12 were negative in the LIPS-G_A_ assay and all were negative in LIPS-G_B_ assay. When the sera collected at 15–18 months were tested, anti-RSV-G IgG antibodies were detected in 5/12 subjects including four (out of 12) against Ruc-G_A_ antigen and one (out of 12) against Ruc-G_B_ antigen. All other samples were negative at follow-up in both assays. Two serum samples obtained at 9 months of age were positive in the LIPS-G_A_ assay but the paired samples collected subsequently were negative when tested against both Ruc-G_A_ and Ruc-G_B_ antigens. 

### 3.7. Reactivity of anti-RSV IgG Antibodies with Mutated Ruc-RSV-mG Antigens

**Figure 9:**
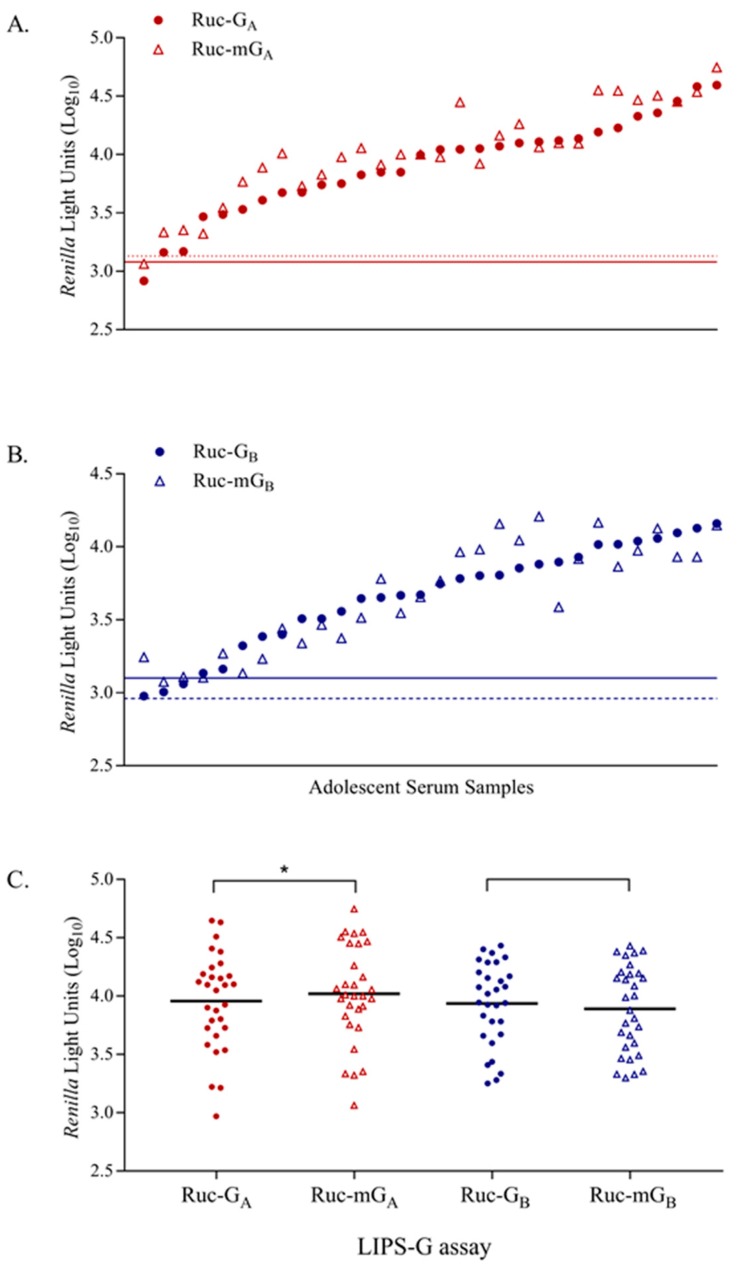
Reactivity of adolescent sera using native and mutated Ruc-G_A_ and -G_B_ antigens. Binding to native versus mutated antigen was compared using the adolescent serum panel. Data were normalized against rabbit anti-FLAG antibody for each antigen comparison; (**A**) Ruc-G_A_ versus Ruc-mG_A_ or (**B**) Ruc-G_B_ versus Ruc-mG_B_. Immunoglobulin depleted serum (IgΔ) was used as the negative control and cutoff values are shown as either solid (native) or dashed (mutated) horizontal red (Ruc-G_A_, Ruc-mG_A_) or blue lines (Ruc-G_B_, Ruc-mG_B_). Results are ranked from low to high based on testing against the Ruc-G_A_ or Ruc-G_B_ antigens and paired with the corresponding result for each subject against the Ruc-mG_A_ or Ruc-mG_B_ antigens, respectively. (**C**) Group RLU responses were plotted for all antigens tested. * indicates significant difference between groups (*p* = 0.0013).

RSV-G protein exists as a membrane bound protein in its native state. However, due to an alternative initiation of translation at the second Met (amino acid 48), a soluble form of G may also be present [27]. To rule out any potential interference of soluble G antigens with results obtained using Ruc-G_A_ or Ruc-G_B_ antigens, Ruc-mG_A_ and Ruc-mG_B_ constructs were generated and used to detect anti-RSV-G_A_ IgG and anti-RSV-G_B_ IgG antibodies. Samples were tested in parallel against native and mutated G antigens (Ruc-G_A_, Ruc-mG_A_, Ruc-G_B_, and Ruc-mG_B_). Human immune globulin (BEI NR-21973), Mabs 1227 and 131-2G, or rabbit antisera (RSV-A2, -18537/G_B_ protein, -G_A_ protein, -LHWT-G_A171-202_ peptide, and -LHWT-G_B171-202_ peptide) had similar reactivities when tested against native versus mutated G proteins. Similarly, when adolescent sera were tested in the LIPS-G_A_ and -mG_A_ assays, 29/30 samples were positive using both Ruc-G_A_ and Ruc-mG_A_ antigens (Figure 9A). In contrast, when tested using Ruc-G_B_ and Ruc-mG_B_ antigens, 27 samples were positive in the LIPS-G_B_ assay whereas all 30 samples were positive in the LIPS-mG_B_ assay (Figure 9B). When the results of testing adolescent sera against Ruc-mG_A_ and Ruc-mG_B_ antigens were compared with the results obtained using the homologous subtype in a PRN test, no correlation was seen. Interestingly, the group mean RLU responses for Ruc-mG_A_ were significantly higher than those seen using Ruc-G_A_ (Ruc-G_A_ = 3.96log_10_ RLU versus Ruc-mG_A_ = 4.02log_10_ RLU with *p* = 0.0013). However, mean responses were similar for the tests using Ruc-G_B_ and Ruc-mG_B_ (Ruc-G_B_ = 3.94log_10_ RLU versus Ruc-mG_B_ = 3.90log_10_ RLU with *p* = 0.4893) (Figure 9C). IgΔ was used as the negative control and cutoffs for a positive response set as follows: 3.08log_10_ RLU for Ruc-G_A_, 3.13log_10_ RLU for Ruc-mG_A_, 3.10log_10_ RLU for Ruc-G_B_, and 2.96log_10_ RLU for Ruc-mG_B_. 

## 4. Discussion

We have described the development of an immunoprecipitation assay using luciferase-tagged RSV-G_A_ and -G_B_ proteins to detect IgG antibodies in serum. Although RT-PCR is routinely used for RSV diagnosis, and this testing is very sensitive for detecting RSV genome, the logistics of sample collection in a timely manner may be complicated. A strategy that relies only upon RT-PCR to identify cases may underestimate the number infected with RSV. When RT-PCR is combined with a serological assay, RSV incidence rates are increased [17]. Commercially available enzyme-linked immunosorbent assay (ELISA) kits that detect anti-RSV-IgG are easy to use and provide rapid results. However, these kits typically use whole virions or lysates from RSV-infected cells and may not be able to discriminate between antibodies elicited by natural infection from those resulting from immunization or immune-prophylaxis. As such, serological assays to detect antibodies against RSV-specific, non-vaccine antigens are needed to support surveillance efforts and help identify RSV-exposed subjects following immunization or immune-prophylaxis. Electrochemiluminescent serological assays using non-vaccine target antigens successfully identified subjects exposed to RSV in the post-immunization period, thus providing support for the practical use of these types of assays [18]. However, reagents for these assays are not commercially available. Similarly, immune profiling by immunoprecipitation of luciferase-tagged proteins (LIPS) allows detection of protein-specific IgG antibodies. Additionally, the use of luciferase-tagged antigens from crude cell lysates is advantageous since protein purification can be time consuming and often results in low yields. Assays that distinguish between the two RSV antigenic subtypes (A and B) may also contribute to our understanding of the role these IgG antibodies may play in determining subsequent risk of RSV re-infection. Lastly, detection of anti-RSV-G IgG antibodies among subjects given RSV-F-only vaccines may help to confirm RSV exposures that support safety and efficacy assessments.

The LIPS-G assays described herein specifically detected IgG antibodies directed against RSV-G_A_ and/or -G_B_ proteins, as shown by murine MAbs, while MAbs directed against RSV-N, RSV-F, PIV3-F, and PIV3-HN failed to bind in either assay. Specificity was further confirmed using mono-specific rabbit antisera induced by immunization with purified RSV-G_A_ protein, or synthetic linear RSV-G_A_ and -G_B_ peptides while rabbit antisera induced by immunization with either purified RSV-F protein, UV-inactivated MV, or UV-inactivated hMPV failed to bind in either assay. Recombinant RSV-G_A_ or RSV-G_B_ proteins blocked binding of human immune globulin in each assay, while recombinant RSV-N protein had no effect, providing additional evidence of assay specificity. 

Interestingly, rabbit antisera generated in response to RSV-G_A_ antigens (i.e., anti-UV-A2, anti-G_A_ protein, and anti-LHWT-G_A_ peptide) reacted with both Ruc-G_A_ and Ruc-G_B_ antigens, although binding was higher against the homologous antigen. Cross-reactivity was likely due to binding within the central conserved region defined by RSV-G amino acids 164-176, which are 100% conserved across subtypes. In contrast, the antiserum to the LHWT-G_B_ peptide only bound to homologous Ruc-G_B_ antigen, suggesting that RSV-G_A_ elicits a more potent and broadly cross-reactive response while RSV-G_B_ antigen may elicit a lower and more focused IgG antibody repertoire. Serum from adolescents 10-14 years of age also contained anti-RSV IgG antibodies that bound both Ruc-G_A_ and Ruc-G_B_ proteins. This was not surprising since it is likely that most children experience multiple, remote RSV exposures of both antigenic subtypes by this age.

In contrast with the cross-reactivity seen using antisera from animals given multiple parenteral doses of RSV virus or protein, anti-RSV-G IgG responses observed in sera from mice experimentally infected intranasally with RSV-A2 or RSV-B1 strains once or twice were subtype specific. Similarly, we documented seroconversion among a small number of infants seronegative for RSV antibodies at 9 months of age who developed subtype specific anti-RSV-G_A_ or -G_B_ IgG responses by 15–18 months of age suggesting they had been infected with either a RSV subtype A or B strain in the interval between sampling. Other investigators have also noted subtype-specific neutralizing antibody and binding responses in serum following primary or early RSV infection in infants and toddlers [19]. The significance of subtype-specific responses against RSV-G_A_ or RSV-G_B_ proteins suggests that these differences early in life could potentially contribute to susceptibility of infants and young children to RSV lower respiratory tract infection (LRTI) if antibody responses against RSV-F are absent, low, or not sustained. 

Although many current RSV vaccines focus primarily on RSV-F protein, the importance of immune responses targeting the other major surface glycoprotein, RSV-G, should not be overlooked. There have been many discoveries about RSV-G in recent years, including its role in mediating attachment to CX3CR1 whereby the virus initiates infection of bronchial epithelial cells, as well as the putative role of secreted RSV-G in modulating the host immune response [7]. Even though RSV-G shows more antigenic diversity than RSV-F, the central conserved region was shown to be an important target for MAb development and antibodies directed to this region were shown to provide effective prophylaxis against RSV infection in animals [10,12,31]. RSV-G antigenic sites outside this central domain can also elicit protective immunity in animals against RSV infection [15]. Since it is apparent that RSV-G can elicit both neutralizing and non-neutralizing antibody responses, a serological assay that determines anti-RSV-G antibody status may be a valuable tool in RSV sero-surveillance. 

There are several limitations to this study. We did not have sequential serum samples from infants or young children before and after documented RSV infection, nor did we have samples from children infected with other respiratory viruses to further define specificity of the assay. RSV-G genes and sequences from known laboratory adapted strains were used in the construction of the luciferase-tagged antigens used for the immunoprecipitation assay. Nevertheless, it may be more relevant to use G proteins derived from contemporary RSV-A and -B viruses to determine if these proteins increase the sensitivity for detecting anti-RSV-G IgG going forward. Lastly, we did not test an antibody that binds to a known conformational site within RSV-G to confirm proper protein folding, since recent work indicates that critical neutralizing anti-G monoclonal antibodies bind in a conformation specific manner [32,33]. Nevertheless, it is well documented that the central conserved region is an immunodominant region within RSV-G that elicits antibodies associated with protection in animals [34]. Both murine monoclonal and rabbit polyclonal antibodies that map to this region were highly reactive against Ruc-G_A_ and/or Ruc-G_B_ antigens, suggesting this critical, protective domain within RSV-G had assumed the proper conformation.

## 5. Conclusions

In conclusion, the LIPS-G_A_ and LIPS-G_B_ assays offer a simple and direct method to detect the immune response to RSV-G protein. These assays are rapid, sensitive, and capable of detecting subtype specific responses. 

## Figures and Tables

**Table 1:** RSV-G primer sequences.

Primer Name	Sequence
RSV-A2-G Forward	AAGGAATTCAACATGTCCAAAAACAAGGACCAACGCACC
RSV-A2-G Reverse	GGGCTCGAGTTAAAGTAACTACTGGCGTGGTGT
RSV-B1-G Forward	AAGGAATTCACCATGTCCAAACACAAGAATCAACGCACT
RSV-B1-G Reverse	GGGCTCGAGGAATAACTAAGCATGTGATTGGGT
